# Effect of acupuncture on episodic memory for amnestic mild cognitive impairment based on hippocampal subregion: study protocol of a randomized, controlled trial

**DOI:** 10.3389/fneur.2025.1536291

**Published:** 2025-07-22

**Authors:** Xinbei Li, Xingying Lu, Juan Ou, Hanqing Lyu, Yongfeng Liu, Jinhuan Zhang

**Affiliations:** ^1^Department of Radiology, The Fourth Clinical Medical College of Guangzhou University of Chinese Medicine/Traditional Chinese Medicine Hospital, Shenzhen, China; ^2^Department of Acupuncture, The Fourth Clinical Medical College of Guangzhou University of Chinese Medicine/Shenzhen Traditional Chinese Medicine Hospital, Shenzhen, China

**Keywords:** amnestic mild cognitive impairment, acupuncture, episodic memory, hippocampal subregions, randomized controlled trial, protocol

## Abstract

**Background:**

The hippocampus is a key brain region for episodic memory, and impairment of episodic memory is the earliest feature of amnestic mild cognitive impairment (aMCI). Preserving the structure and function of the hippocampus plays a key role in preventing progression to Alzheimer’s disease (AD). Therefore, early intervention is crucial, and acupuncture could significantly improve episodic memory in aMCI, but the mechanism is not clear. Accordingly, this experiment aims to explore the mechanism of acupuncture to improve episodic memory in aMCI based on the hippocampal subregion.

**Methods:**

A randomized, controlled, blinded research will be performed. 150 aMCI participants will be assigned to the verum acupuncture group, sham acupuncture group, and waiting group; 50 healthy controls will also be recruited. Patients in the acupuncture group will receive real acupuncture treatment or placebo acupuncture three times per week, 36 sessions over 12 consecutive weeks in total. Patients in the waiting group will receive the same verum acupuncture treatment for compensation after the trials are finished. The primary outcome will be the auditory-verbal learning test. Secondary outcomes will include Montreal Cognitive Assessment (MoCA), Mini-Mental State Examination (MMSE), Hamilton Depression Scale (HAMD), and functional magnetic resonance imaging data. Outcomes will be measured at baseline and 12 weeks after randomization. Repeated measurement analysis of variance will be used to explore the intervention effect.

**Discussion:**

This protocol will provide theoretical support for the neural mechanisms by which acupuncture improves episodic memory in aMCI. We hope that this study will provide objective evidence and offer alternative treatment options for the early treatment of aMCI.

**Clinical trial registration:**

ChiCTR2400084308.

## 1 Introduction

Mild cognitive impairment (MCI) refers to a state in which memory loss is greater than aging while cognitive function still remains, a transitional stage between normal cognition and Alzheimer’s disease (AD) (1). Recent epidemiological investigations indicate that the prevalence of mild cognitive impairment (MCI) in China is approximately 15.5%, corresponding to an estimated 38.77 million individuals (2). Advanced age constitutes a significant risk factor for MCI (3). According to the United Nations Department of Economic and Social Affairs, Population Division (4), the global population aged 65 and over is projected to account for nearly 12% by 2030. By 2050, this figure is expected to rise to 16%. Specifically, by 2050, the global population aged 65 and above is anticipated to reach 1.8 billion, constituting 16% of the total population. Meanwhile, the global population aged 60 and above is projected to reach 2 billion by 2050, accounting for 18% of the total population. Consequently, the number of individuals with MCI is expected to exhibit an increasing trend. MCI can be categorized into three subtypes: amnestic mild cognitive impairment (aMCI), multiple domain MCI and single non-memory domain MCI (e.g., language and visuospatial) (5). Previous studies have shown that patients with aMCI are more likely to progress to AD compared to similarly aged and cognitively normal individuals and those with non-aMCI (naMCI) (6, 7). Therefore, it is particularly important to diagnose and intervene early in aMCI and to prevent it from worsening during the aMCI stage.

In recent years, the disease-modifying therapies (DMTs), which aim to restrain the neuropathological progression of AD, have been evolving rapidly. Unfortunately, most drugs based on DMTs have not been put into clinical application or are still in the clinical trial phase (8). Researchers have found that punicalagin in pomegranate may target the underlying mechanisms of MCI and enhance therapeutic outcomes. However, there is no nutritional health therapy with regard to pomegranate to suppress it (9). Therefore, it is crucial to find a safe and effective therapy for the treatment of aMCI.

Recently, acupuncture, as a safe and reliable non-drug therapy, has been fully confirmed for its efficacy in the treatment of cognitive impairment (10–14). Our previous randomized controlled trial (RCT) also proved it can alleviate cognitive symptoms (15). However, the mechanism of acupuncture treating aMCI has not been revealed.

With the development of magnetic resonance imaging (MRI) technology, several studies have shown that the structure and function of specific brain regions are altered in patients with aMCI (16–18). Notably, the hippocampus, as the hub of regions dealing with episodic memory (19), is closely related to the development of aMCI (20). Episodic memory is a declarative memory in which the brain combines the content of an event that triggers an individual’s emotional or ideological awakening with spatio-temporal information to encode and store it (21). Episodic memory belongs to the category of long-term memory, which is an advanced cognitive function possessed by humans. The researchers found that during the formation, object “what,” spatial “where” and temporal “when” of episodic memory were transmitted to the hippocampus through different information flow paths for integrated coding (22) Researchers found that there is a decrease in the volume of the hippocampus and abnormal hippocampal dynamic functional connectivity (dFC) in aMCI, compared to healthy people (23, 24).

Therefore, we hypothesize that acupuncture could enhance episodic memory in aMCI by remodeling hippocampal subregional connectivity and volume, thereby offering a mechanistic basis for its clinical application. Then we designed a randomized controlled trial with the aim of (1) evaluating the efficacy and safety of acupuncture in improving episodic memory in aMCI patients and (2) exploring the structural and functional changes of hippocampal subregion underlying acupuncture’s therapeutic effects.

## 2 Methods

### 2.1 Study design

**Figure 1:**
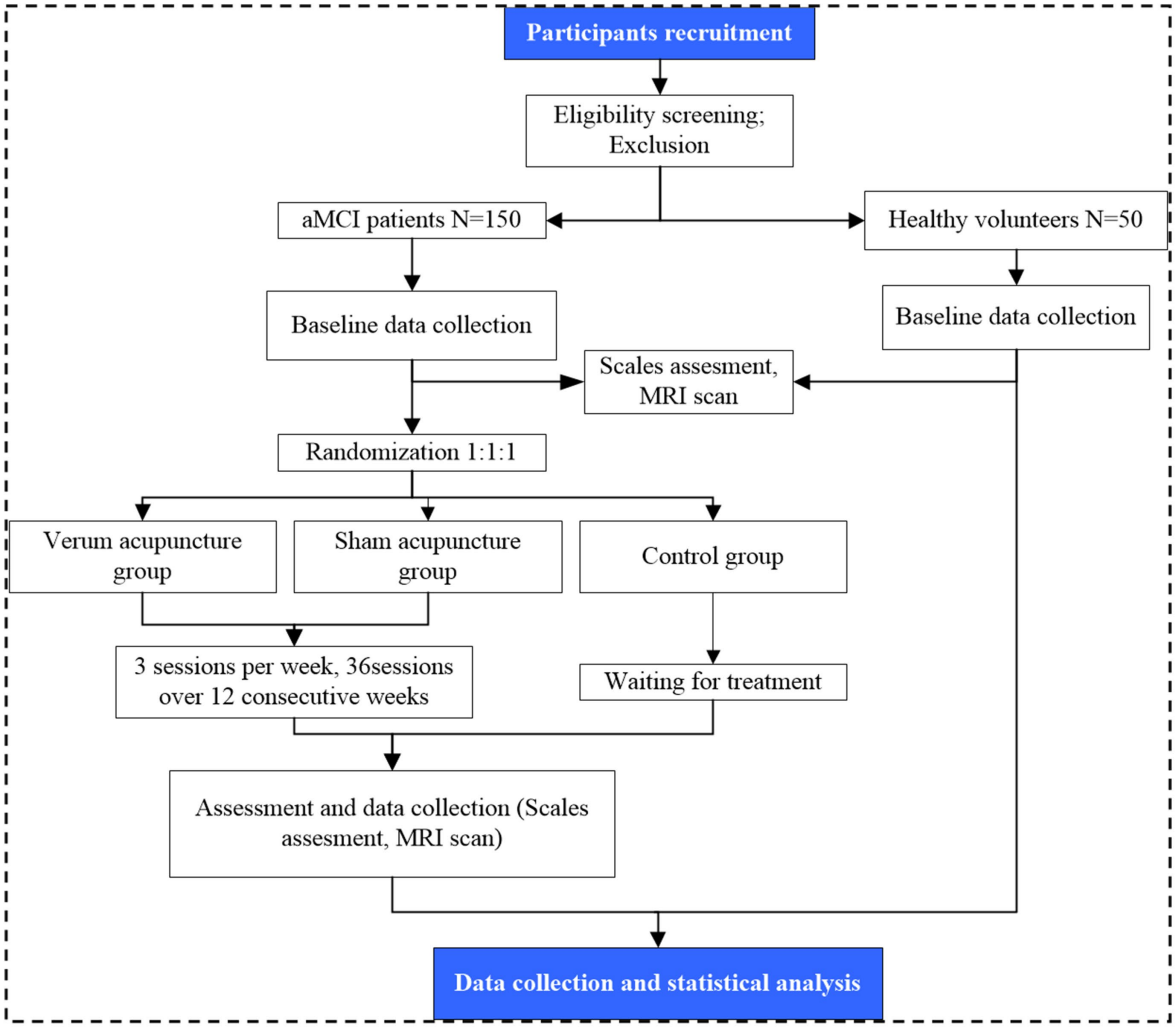
Flow chart of the trial procedures.

This is a prospective, single-center, three-armed, parallel-designed, assessor-blinded, randomized controlled trial. A total of 150 participants with aMCI and 50 healthy controls (HCs) will be enrolled. The 150 eligible patients will be randomly assigned to the verum acupuncture group, the sham acupuncture group, and the waiting group in a ratio of 1:1:1. Healthy volunteers will serve as a comparison group for baseline comparisons. The enrollment schedules, interventions, and assessments are summarized in Table 1. A study flowchart is provided in Figure 1.

**Table 1:** Schedule of enrollment, intervention, and assessments.

Item	Study period				
	aMCI				Healthy volunteers
	Baseline	Treatment phase			Baseline
	0 week	Week 1	→	Week 12	0 week
Enrollment					
Inclusion criteria	×				×
Exclusion criteria	×				×
Informed consent	×				×
Intervention					
Verum acupuncture group		×	×	×	
Sham acupuncture group		×	×	×	
Control group					
Outcome measurement					
Clinical features					
AVLT-H	×			×	×
MoCA	×			×	×
MMSE	×			×	×
HAMD-17	×			×	×
fMRI					
3DT1WI	×			×	×
T2WI	×			×	×
Flair	×			×	×
rs-fMRI	×			×	×
DTI	×			×	×
Safety					
Adverse events		×	×	×	

### 2.2 Sample size

The sample size for MRI studies is typically determined by practical constraints such as scanning time, cost, and effect size, so there is no unified standard. Desmond et al. conducted a statistical efficiency analysis based on the estimation of sample size in MRI studies, indicating that approximately 12 participants can achieve 80% power in typical activation at the voxel level (25). After considering that increasing the sample size could significantly enhance the stability of the results and accounting for a certain dropout rate, we therefore decided that each group will include 50 participants, totaling 150 aMCI patients and 50 healthy controls.

### 2.3 Recruitment

Participants with aMCI and HCs for this study will be recruited from Shenzhen Traditional Chinese Medicine Hospital. All potential participants will be thoroughly briefed on the objectives, its procedures, potential benefits, and associated risks of the study protocol. Before enrollment, they will be asked to voluntarily sign an informed consent form, signifying their understanding and agreement to participate in the study.

### 2.4 Eligibility criteria

#### 2.4.1 Inclusion criteria

Patients of aMCI meet the following criteria: (1) diagnosis based on the aMCI diagnostic criteria proposed by Petersen (26); (2) right-handedness; (3) age 50–75 years without limitation of gender; (4) Objective Memory Impairment confirmed Auditory Verbal Learning Test Fifth Trial (AVLT_N5) score > 1 standard deviation (SD) below the age-adjusted normative mean; (5) self-reported memory impairment or cognition complaints by caregivers, with a disease course > 6 months.

Healthy controls meet the following criteria: (1) cognitive integrity confirmed by Mini-Mental State Examination (MMSE) score ≥27 and absence of subjective cognitive complaints; (2) right-handedness; (3) age 50–75 years without limitation of gender.

#### 2.4.2 Exclusion criteria

Patients with aMCI who meet any of the following criteria will be excluded: (1) a history of or current diagnosis of neurological or neuropsychiatric disorders that may impair cognitive function (e.g., stroke, epilepsy, major depressive disorder, schizophrenia, or neurodegenerative diseases such as Parkinson’s disease); (2) recent participation in therapeutic interventions (e.g., acupuncture or pharmacotherapy) within the past 4 weeks or concurrent enrollment in other clinical trials; (3) inability to complete neuropsychological assessments or undergo MRI examination due to severe sensory deficits (e.g., aphasia, blindness, hearing loss) or MRI contraindications; (4) clinically significant unstable systemic conditions (e.g., cardiac, hepatic, renal or respiratory diseases); (5) systemic diseases or comorbidities associated with cognitive impairment (severe anemia, HIV infection, untreated thyroid dysfunction, syphilis, alcohol and drug addiction, or anthrax).

Healthy controls meeting following criteria will be excluded: (1) a history of or current diagnosis of neurological, neuropsychiatric, or systemic disorders associated with cognitive impairment (epilepsy, Parkinson’s disease, severe anemia, HIV infection, alcohol or drug addiction, syphilis, or thyroid dysfunction); (2) contraindications to MRI examination; (3) clinically significant unstable systemic conditions (e.g., cardiac, hepatic, renal or respiratory diseases); (4) participation in other clinical trials within the past 6 months.

### 2.5 Randomization, allocation concealment, and blinding

After baseline evaluation, the eligible patients of aMCI will be randomly assigned to a verum acupuncture group, a sham acupuncture group, and a waiting group. The randomly assigned sequences will be generated using SPSS. The independent research assistant will inform eligible participants of their allocation results by telephone. We could not establish the blinding of participants, acupuncturists, or intervention supervisors to the assigned treatment, but outcome assessors and data statisticians will be blind to the grouping assignment.

### 2.6 Interventions

The interventions are compliant with the Consolidated Standards of Reporting Trials (27) and the Standards for Reporting Interventions in Clinical Trials of Acupuncture (28).

#### 2.6.1 Verum acupuncture group

**Figure 2:**
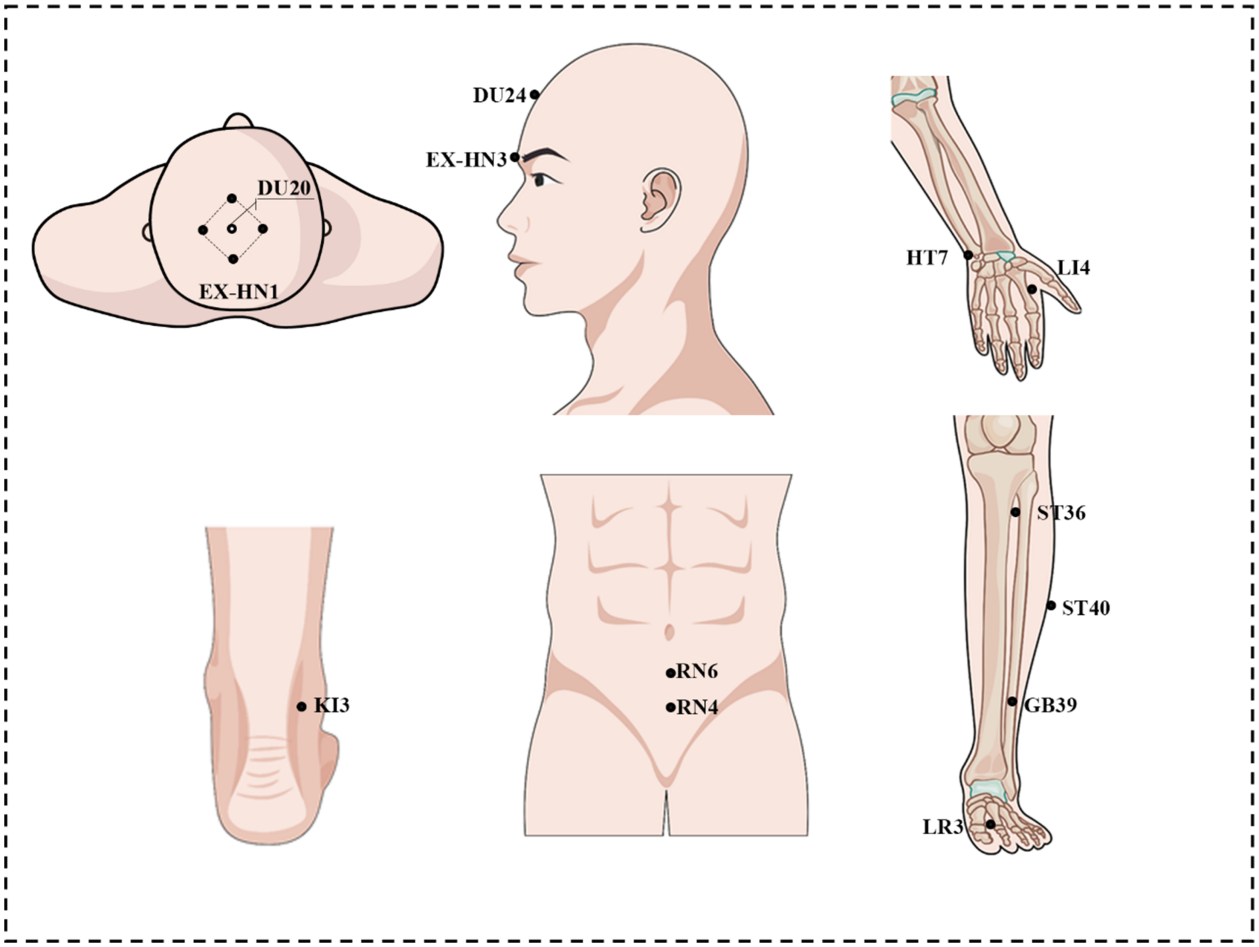
Location of selected acupoints.

Patients allocated to the verum acupuncture group will receive treatment with needles inserted at the pre-specified acupuncture points. The acupoints include Baihui (GV20), Sishencong (EX-HN1), Shenting (GV24), Yintang (EX-HN3), Qihai (CV6), Guanyuan (CV4), bilateral Hegu (LI4), Shenmen (HT7), Zusanli (ST36), Fenglong (ST40), Xuanzhong (GB39), Taixi (KI3), and Taichong (LR3). Acupoint locations were determined according to the WHO Standard Acupuncture Point Locations in the Western Pacific Region (29) and are illustrated in Figure 2 and Table 2. The selection of these acupoints was based on clinical experience and previous experimental evidence. Prior clinical validation from our RCT (15) demonstrated cognitive improvement with the identical acupoint protocol. Manipulations including twirling, lifting, and thrusting will be performed on all needles for at least 30 min to reach De qi (a compositional sensation including soreness, numbness, distention, and heaviness), which is considered an essential component for acupuncture efficacy (30).

**Table 2:** Locations of acupoints.

Acupoints	Locations	Insert depth
Baihui (GV20)	On the head, 5 B-cun superior to the anterior hairline, on the anterior median line	0.5 ~ 0.8 cun
Sishencong (EX-HN1)	On the vertex, 1 cun from the front, back, left, and right to GV20, common 4 points	0.5 ~ 0.8 cun
Shenting (GV24)	On the head, 0.5 B-cun superior to the anterior hairline, on the anterior median line	0.5 ~ 0.8 cun
Yintang (EX-HN3)	At the forehead, at the midpoint between the two medial ends of the eyebrow	0.3 ~ 0.5 cun
Qihai (CV6)	On the lower abdomen, 1.5 B-cun inferior to the centre of the umbilicus, on the anterior median line	1 ~ 1.5 cun
Guanyuan (CV4)	On the lower abdomen, 3 B-cun inferior to the centre of the umbilicus, on the anterior median line	1 ~ 1.5 cun
Hegu (LI4) (bilateral)	On the dorsum of the hand, radial to the midpoint of the second metacarpal bone	0.5 ~ 1 cun
Shenmen (HT7) (bilateral)	On the anteromedial aspect of the wrist, radial to the flexor carpi ulnaris tendon, on the palmar wrist crease	0.3 ~ 0.5 cun
Zusanli (ST36) (bilateral)	On the anterior ascpect of the leg, on the line connecting ST35 with ST41, 3 B-cun inferior to ST35	1 ~ 2 cun
Fenglong (ST40) (bilateral)	On the anterolateral aspect of the leg, lateral border of the tibialis anterior muscle, 8 B-cun superior to the prominence the lateral malleolus	1 ~ 1.5 cun
Xuanzhong (GB39) (bilateral)	On the fibular aspect of the leg, anterior to the fibula, 3 B-cun proximal to the prominence of the lateral malleolus	0.5 ~ 0.8 cun
Taixi (KI3) (bilateral)	On the posteromedial aspect of the ankle, in the depression between the prominence of the medial malleolus and the calcaneal tendon	0.5 ~ 0.8 cun
Taichong (LR3) (bilateral)	On the dorsum of the foot, between the first and second metatarsal bones, in the depression distal to the junction of the bases of the two bones, over the dorsalis pedis artery	0.5 ~ 0.8 cun

* B-cun: proportional bone (skeletal) cun.

#### 2.6.2 Sham acupuncture group

**Figure 3:**
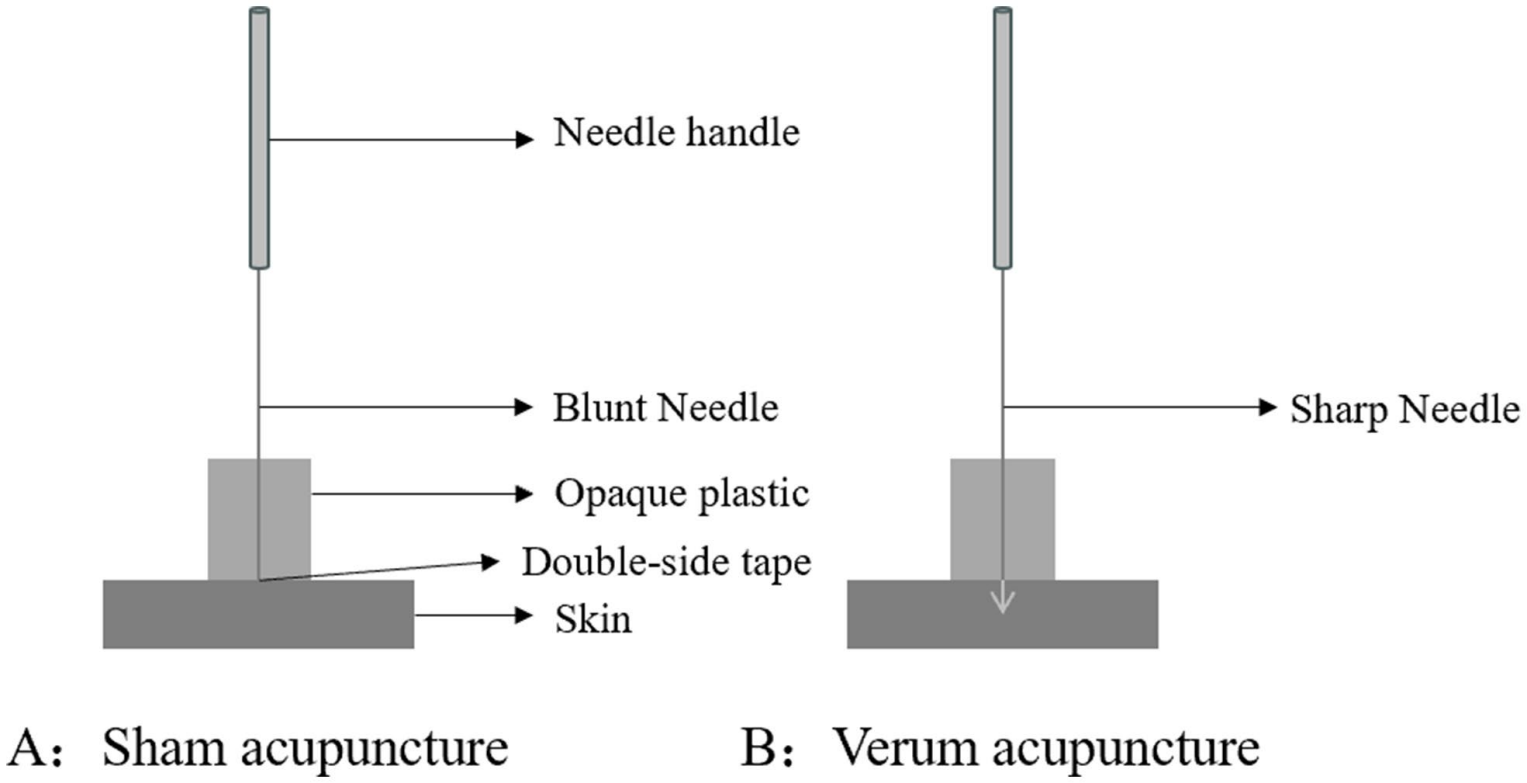
Park sham acupuncture device.

The acupuncturist will use a placebo needle in the sham acupuncture group. The placebo needle, whose tip is blunt, is designed so that participants will have a pinprick-like sensation when it touches the skin, while the needle will not penetrate the skin and appears to be inserted (Figure 3). The same acupoints as those in the verum acupuncture group are selected for placebo needle stimulation to avoid a site-specific effect of acupuncture.

#### 2.6.3 Waiting group

Patients in the WG will not receive acupuncture treatment during the observation period, and they will be provided 36 acupuncture sessions for free after observation.

#### 2.6.4 Healthy group

The healthy participants will not receive any treatment.

### 2.7 Clinical outcome measures

All patients will receive following scale assessment twice: in 12–24 h before the first acupuncture and in 24–72 h after the last acupuncture. Healthy volunteers will receive the assessment at baseline.

#### 2.7.1 Primary outcomes

Episodic memory will be evaluated using the Auditory Verbal Learning Test-Huashan version (AVLT-H), which includes immediate recall (AVLT_N1-N3), 5 min delayed recall (AVLT_N4), 20 min delayed recall (AVLT_N5), cued recall (AVLT_N6), and recognition test (AVLT_N7). With a good reliability and validity in evaluating episodic memory of patients with aMCI (31), AVLT-H is used to assess the short-term delayed free recall and the long-term delayed free recall. The participant will be asked to learn and repeat a list of 12 words three times, with a short-delayed recall after an interval of 3–5 min, and a long-delayed recall, cue recall and identification after an interval of 20 min. The most sensitive evaluation index is the score of long-delay recall and identification. The higher the score, the better the memory function.

#### 2.7.2 Secondary outcomes

The secondary outcomes include the following assessments: (1) Montreal Cognitive Assessment (MoCA), an accurate cognitive screening tool for use in general practice settings; (2) Mini-Mental State Examination (MMSE), a test used to assess overall cognitive function in patients with aMCI; (3) Hamilton Depression Scale (HAMD), a scale used to evaluate the emotional status of patients.

#### 2.7.3 MRI outcomes

All patients will receive MRI scans twice: in 12–24 h before the first acupuncture and in 24–72 h after the last acupuncture. Healthy volunteers will undergo MRI scan at baseline. The 3D-T1 MRI scans will be conducted with a 3.0 T MRI scanner (Siemens MAGNETOM Prisma 3.0 T). The parameters for the T1 images for dataset 1 are as follows: repetition time (TR) = 2,200 ms, echo time (TE) = 2.45 ms, flip angle = 8°, field of view = 256 × 256 mm^2^, matrix size = 256 × 256, voxel size = 1 × 1 × 1 mm^3^, and slices = 175.

Resting-state functional MRI (rsfMRI) data will be collected within 7 min using the following parameters: epetition time = 2,000 ms, echo time = 30 ms, flip angle = 90°, field of view = 220 × 220 mm 2, matrix = 64 × 64, slice bb thickness = 3.2 mm, slice number = 37, and 240 volumes.

### 2.8 Statistical analysis

Statistical analyses will be performed using SPSS software (Version 27.0, IBM Corp.), with a two-tailed *p*-value <0.05 being considered statistically significant. Normally distributed continuous variables will be expressed as mean ± standard deviation, with between-group comparisons being analyzed by independent samples t-test or one-way ANOVA (as appropriate), and within-group comparisons by paired t-test or repeated measures ANOVA. Non-normally distributed data will be presented as median (interquartile range) and analyzed using Wilcoxon rank-sum test. Categorical variables will be reported as frequencies (percentages), with between-group differences being assessed by chi-square test. In clinical between-group multiple comparisons, all *p*-values will be adjusted using the false discovery rate (FDR, Benjamini-Hochberg procedure) to control the risk of false-positive results (11).

Neuroimaging data will be processed using the DPABI V5.4 toolkit. Voxel-based morphometry (VBM) will be implemented to quantify volumetric characteristics of hippocampal subfields, while dynamic functional connectivity (aDC) analysis will be employed to characterize time-varying functional interactions between hippocampal subregions and other brain areas. Covariates including gender, age, educational attainment, and total intracranial volume will be regressed out during preprocessing. Statistical parametric maps will undergo Gaussian Random Field (GRF) correction for multiple comparisons at the cluster level. To detect voxel-wise alterations in hippocampal subfield functional connectivity (FC), mixed-effects models will be constructed to assess group differences, with interaction effects rigorously tested through permutation testing. All statistical outcomes will subsequently undergo False Discovery Rate (FDR) correction to control family-wise error across the entire analytical pipeline.

### 2.9 Safety measurements

Any possible accidental injuries caused by this study will be prevented as much as possible, the relevant medical expenses will be covered by the research project team, and the participants will receive certain financial compensation. Any unexpected adverse events during the intervention period will be monitored and reported to a research assistant. Causality in relation to the acupuncture intervention and the severity of adverse events will be analyzed. Serious adverse events will be reported to the ethics committee.

## 3 Discussion

The present study suggests that acupuncture improves episodic memory function in aMCI by remodeling the structure and function of hippocampal subregions, which is consistent with the findings of previous studies (11).

In the theory of Traditional Chinese Medicine (TCM), Amnestic Mild Cognitive Impairment (aMCI) is categorized under the condition of “forgetfulness.” The etiology of this disorder is attributed to the deficiency of cerebral essence, the lack of nourishment to the primal spirit, stagnation of qi and dampness, and the failure of clear yang to ascend, which collectively lead to progressive memory loss. Based on clinical experience and previous experimental evidence, the acupuncture prescription has been formulated to address these issues. The prescription includes the following acupoints: Baihui (GV20), Sishencong (EX-HN1), Shenting (GV24), Yintang (EX-HN3), Qihai (CV6), Guanyuan (CV4), bilateral Hegu (LI4), Shenmen (HT7), Zusanli (ST36), Fenglong (ST40), Xuanzhong (GB39), Taixi (KI3), and Taichong (LR3). This selection of acupoints aims to replenish the cerebral essence, nourish the primal spirit, regulate the flow of qi, resolve phlegm, and thereby enhance cognitive function. Moreover, patients with MCI who received acupuncture at acupoints similar to those used in our study (Sishencong EX-HN1, Yintang GV29, Neiguan PC6, Taixi KI3, Fenglong ST40, and Taichong LR3) exhibited improved cognitive function (32). Corresponding rs-fMRI findings demonstrated strengthened functional connectivity within a cognition-associated network post-intervention, specifically within key regions such as the insula, dorsolateral prefrontal cortex, hippocampus, thalamus, inferior parietal lobule, and anterior cingulate cortex (32). In addition, acupuncture at Taichong (LR3) and Hegu (LI4) has been shown to activate prefrontal and hippocampal regions – critical hubs for memory consolidation – in both MCI and Alzheimer’s disease populations (33).

The hippocampus, as an important brain region in the central nervous system, is involved in learning and memory storage (34). However, it is recognized that the cytoarchitecture of hippocampus is not homogeneous (35). A growing body of research has found that analyzing of hippocampal subregions better detects subtle changes in hippocampal structure, more accurately discerns the cognitive relevance of these changes (36, 37). Therefore, it is necessary and reasonable to explore acupunctures based on hippocampal subregions to improve episodic memory functions. Modern studies have demonstrated that acupoints such as Baihui (GV20), Shenting (GV24) and Sishencong (EX-HN1) can improve cognitive function (38–40), which aligns with the traditional TCM theory of nourishing the brain and enhancing memory. Animal experiments further confirm that electroacupuncture at Baihui (GV20) and Shenting (GV24) can affect proteomic changes in the hippocampus of rats with cognitive impairment, increase the basic synaptic transmission efficiency and synaptic plasticity of the hippocampal CA3-CA1 circuit, thereby improving learning and memory ability (38, 39). Through this tailored acupuncture approach integrating classical theory with contemporary neuroimaging evidence, further exploration of the neural mechanisms of acupuncture on episodic memory in aMCI subregions in humans is necessary.

Furthermore, in the design of using sham acupuncture as the control group, we will use blunt needles at acupoints rather than non-acupoints. The primary reason is that this study aims to verify the therapeutic mechanism of acupuncture on episodic memory in aMCI patients, not to validate acupoint specificity. Applying blunt needles at acupoints for SA aims to maximally simulate the VA treatment environment (including the operation site, doctor-patient interaction, and sensory experience), thereby more strictly controlling the placebo effect and reducing the risk of unblinding for patients. In fact, regarding acupoint selection, in addition to the 362 conventional acupoints, there are numerous extraordinary acupoints distributed throughout the body. Therefore, it is difficult to find inactive superficial acupoints. Concerning needle insertion methods, both superficial needling and needle withdrawal create some nerve stimulation in the area. This stimulation can then affect related parts of the brain, leading to what are called “edge contact effects,” which is not an inactive placebo (41, 42). Therefore, this finding does not mean that acupoints are unimportant, it shows that the we must carefully consider the placebo effect in acupuncture research.

The main advantages of this study are as follows: first, aMCI patients will be compared with healthy individuals to identify pathological changes in hippocampal subregions. Meanwhile, we will explore how acupuncture reverses hippocampal neuroplasticity alterations in aMCI. The mechanism of acupuncture’s efficacy is based on a rigorous experimental design, ensuring the reliability of the results.

The limitations of this study include the potential difficulty in implementing blinding for sham acupuncture. Additionally, due to the large number of acupuncture points used, it is challenging to identify the specific mechanisms of individual points. Moreover, given the long treatment course, there will be a considerable risk of patient dropout before completing the entire course.

In conclusion, the efficacy of acupuncture in improving episodic memory in patients with amnestic mild cognitive impairment (aMCI) will be further confirmed. This improvement will be achieved by modulating the structural and functional connectivity of hippocampal subregions, leading to enhanced episodic memory function. We hope this can provide an alternative treatment option for aMCI, laying the foundation for personalized precision treatment and outcome prediction.
